# Plural Embodiment(s) of Mind. Genealogy and Guidelines for a Radically Embodied Approach to Mind and Consciousness

**DOI:** 10.3389/fpsyg.2018.02204

**Published:** 2018-11-29

**Authors:** Mauro Ceruti, Luisa Damiano

**Affiliations:** ^1^Department of Communication, Arts and Media, Libera Università di Lingue e Comunicazione (IULM) University, Milan, Italy; ^2^Research Group on Epistemology of the Sciences of the Artificial, Department of Ancient and Modern Civilizations, University of Messina, Messina, Italy

**Keywords:** autonomy, autopoiesis, constructivism, cybernetics of networks, (radical) embodiment, enaction, experimental epistemology, synthetic modeling

## Abstract

This article focuses on a scientific approach to the study of cognition that Warren McCulloch introduced in the era of cybernetics as “experimental epistemology.” In line with recent attempts to highlight its contribution to cognitive science and AI, our article intends to draw attention to its unexplored influence on contemporary embodied approaches to the investigation of mind and consciousness. To this end, we will survey a series of models of cognitive systems genealogically related to the McCulloch-Pitts networks-based modeling approach, i.e., von Foerster’s model of the biological computer, the Maturana-Varela model of the autopoietic system, and Varela’s model of emergent selves. Based on examination of the relevant aspects of these models, we will argue that they offered the McCulloch-Pitts “cybernetic of networks” a coherent methodological and theoretical line of development, complementary to the well-known computationalist one. As we will show, this alternative evolutionary line empowered the biological orientation of McCulloch’s experimental epistemology, laying foundations for contemporary “radically embodied” approaches to mind and consciousness – in particular the Thompson-Varela approach. We will identify the heritage of this tradition of inquiry for future research in cognitive science and AI by proposing guidelines that synthetize how its methodological and theoretical insights suggest taking into account the role(s) played by the biological body in cognitive processes – consciousness included.

## Introduction

“Experimental epistemology” is the designation given to one of the most original scientific projects in the era of cybernetics. Neurophysiologist Warren McCulloch, the project’s creator, introduced this label to differentiate his undertaking from other contemporary scientific approaches to the study of cognition. McCulloch’s approach emphasized merging neurophysiological and philosophical research, specifically by employing a rigorous “experimental” approach in neurophysiology to revisit the questions traditionally asked by philosophy of knowledge – i.e., “epistemology” in the classical sense. In concrete terms, this meant focusing the scientific inquiry on complex dimensions and properties of thought, as defined by philosophical investigations, and explaining them in terms of plausible neurophysiological mechanisms, based on experimental research.

Beginning in the 1940s, McCulloch used these programmatic bases to develop experimental epistemology as a trans-disciplinary science of mind focusing on studying, as legitimate objects of experimental and quantitative research, all mental processes – consciousness included.

The methodological approach was genuinely cybernetic. McCulloch recognized that traditional analytic procedures are indispensable to accessing anatomical and neurophysiological structures of the brain, but that they cannot appropriately support the exploration of its functions. Understanding how the brain generates cognitive processes requires studying its mechanisms in operation, which demands a research method centered not on analysis, but on synthesis. With other pioneers of cybernetics, McCulloch saw this new methodological approach in the “synthetic method” emerging from the proto-cybernetic movements of the beginning of the 20th century (Cordeschi, 2002). Like other cyberneticists, he interpreted it as complementing the traditional analytical method (Craik, 1943), and implemented it along the lines of the “understanding by building” methodological slogan that, still today, guides frontier research in AI and the other sciences of the artificial (Pfeifer and Scheier, 1999; Damiano and Stano, 2018a,b). In short, the core idea is to seek the mechanisms underlying natural complex processes through scientifically informed attempts to artificially re-create those processes. In the case of the synthetic study of mental processes, this means elaborating theoretical hypotheses on how the brain might generate the target processes, detailing the hypotheses in terms of plausible mechanisms, and testing the hypotheses by embodying these mechanisms in (possible or actual) artifacts apt to produce the target processes.

McCulloch’s work in experimental epistemology was groundbreaking. As recent attempts to highlight its contribution to science show, his approach to the modeling of brain activity, developed with Walter Pitts in terms of networks of idealized neurons, has served as scaffolding for some of the most generative fields of contemporary science – cognitive science, AI, computer science, neuroscience and neural nets, among others (e.g., Kay, 2001; Dupuy, 2009).

Drawing on these lines, our article intends to contribute to illuminating the influence that McCulloch’s experimental epistemology has had, and still can have, on scientific research on mind and consciousness. Yet, unlike the most widely published readings of the relevance of McCulloch’s work in this field, we will not concentrate on its impact on computationalist, that is, classical cognitive science and AI. Instead, we will bring into focus another line of development of McCulloch’s experimental epistemology, usually neglected despite its influence on the emergence of embodied cognitive science – in particular, its radical lines.

Starting in the 1950s, McCulloch’s work contributed to the rise to a set of interconnected research lines – i.e., second order cybernetics, autopoietic biology, Varelian enaction – engaged in proposing an alternative to computationalism. As we will show, these strands have genealogical, theoretical and methodological connections with McCulloch’s line of inquiry, and share a series of elements uniting them in a coherent research tradition – the tradition of experimental epistemology. In fact, the proponents of these research approaches defined their investigations through the notion of epistemology – sometimes “experimental epistemology” (Maturana, 1974; Varela, 1986). Aligning with McCulloch’s approach, they combined neurophysiological and philosophical research, as well as the analytic and synthetic methods. Furthermore, they used this approach to rework McCulloch’s modeling of cognitive systems in terms of networks, based on a principle of continuity between life and cognition. This produced a progression of models that, redrawing the McCulloch-Pitts networks based on biological insights, generated early, radically embodied approaches to the study of mind and consciousness.

This article intends to reconstruct and analyze the main phases of this unexplored line of development of experimental epistemology, and define guidelines that, according to its radicalization of McCulloch’s idea of “embodiments of mind,” can spur contemporary embodied cognitive science on to look for non-trivial accounts of the role(s) played by the biological body in cognition.

## Two Co-Emerging Paradigms

### The McCulloch-Pitts Cybernetics of Networks

When McCulloch introduced the label “experimental epistemology,” he intended to designate a research tradition to which he considered he belonged. One of the last representatives of this tradition, which McCulloch dated back to physiologist Rudolph Magnus, was Dusser de Barenne, who was McCulloch’s guide in neurophysiological inquiry during the second half of the 1930s (McCulloch, 1961a,b). From de Barenne, McCulloch inherited the postulate of the primacy of the “physiological *a priori*,” conceived of in terms of a Kantian synthetic *a priori* hard-wired in the nervous system. Based on this idea, McCulloch worked with de Barenne’s group on mapping circuit action in the brain. The resulting wiring diagrams led him to a key hypothesis: from perception to consciousness, all cognitive processes are generated by neuronal impulses moving on brain-pathways.

This thesis is the core of the McCulloch-Pitts synthetic approach to modeling brain activity. In the late 1930s, based on Alan Turing’s studies on machine intelligence and Claude Shannon’s application of Boolean algebra to electric circuits, McCulloch began building the basic theoretical image of this approach. Neurons can be characterized as computational units that define their “all-or-none” – 0 or 1 – state by performing logic calculations on the 0/1 signals received from the other neurons, and, through these operations, neuronal activity generates, in the brain-pathways, flowing sequences of 0 and 1 s, which are constitutive of all mental processes.

In 1941, McCulloch, with Pitts, started transforming this picture into a model describing how the brain might do logic processing neuro-biologically. The resulting article – *A logical calculus of ideas immanent in nervous activity* (McCulloch and Pitts’, 1943) – can be read as follows. Shannon demonstrated the possibility of digital computing machines, showing how Boolean functions can be implemented in electric circuits. McCulloch and Pitts, by drawing artificial networks of neurons, demonstrated that the brain can be conceived of as a machine of this kind – a Turing machine. Indeed, according to the 1943 McCulloch-Pitts modeling, neurons are functional units analogous to Boolean logic operators (AND, OR, etc.), and compute their states by applying the rules of Boolean logic to their input signals. When these “compute-and-fire” units are interconnected in networks, they are able to implement complex Boolean functions. Related computation activity generates the logic propositions that are constitutive of mental life – perception, ideas, purposes, etc.

The McCulloch and Pitts’ approach had major consequences. From the scientific point of view, the most general was that all mental process – from perception to learning, from reasoning to consciousness – could be conceived of in terms of mechanisms. The McCulloch-Pitts networks released mind from the “ghostly” status it had assumed in physiology, and reintegrated it among the objects of experimental and quantitative investigation. Indeed, from a philosophical point of view, what McCulloch and Pitts were proposing was a viable alternative to the *res cogitans* postulated by René Descartes. They were modeling mind not as a substance, but as a process. More specifically, mind was an intrinsically dynamic and collective entity, “immanent” in – distributed in or emerging from – the computational activity of a population of neurons. Mind, instead of being a “ghost in the [cerebral] machine” (Ryle, 1949), was the functioning machine itself, that is, a logic machine realized as a network of binary operations of Boolean computation.

The main thesis of the 1943 article, conveyed in McCulloch’s expression “embodiments of mind,” was this: All mental life is immanent in the computational activity of networks of neurons. Consciousness – like other cognitive processes – is computational processing performed by neuronal networks and has to be understood computationally.

In the evolution of the nascent cognitive science and AI, this thesis played a crucial role, grounding at least two influential research paradigms. Chronologically, the first was the computationalist paradigm, which strengthened the McCulloch-Pitts approach’s connection to traditional theory of knowledge. The second was the constructivist paradigm, which reinforced its connection to biology.

### Mind as an Input–Output Machine. Toward Computationalism

In McCulloch and Pitts’ (1943) article, the description of the brain as a computational device was proposed in the form of an equivalence theorem that identified McCulloch-Pitts networks with universal calculators. Indeed, in this work, artificial networks were characterized as systems that, like Turing machines, transform inputs into outputs based on classical propositional logic laws. The main point of the McCulloch-Pitts theorem was that, “with a proper circuit,” the networks “could compute any computable number, and hence could reach any conclusion given by a finite set of premises […]” (McCulloch, 1965).

Almost immediately, this thesis attracted the interest of specialists working on the foundations of computer science. The 1943 McCulloch–Pitts synthetic model of neuronal activity was included among the official theoretical sources of the functional analogy between brain and computer that, starting in 1946, progressively grounded the nascent computationalist cognitive science and AI. Nearly a decade later, the related research paradigm described cognitive processes in approximately this way: The brain receives information through its senses, which work as input devices. It processes the information through neuronal operations, consisting of computations operated on the syntactic aspect of physical symbols. Through these operations, the brain produces internal representations of the outside world, and generates plans of effective actions as motor system outputs.

Although it clearly converged with this theoretical landscape, the McCulloch and Pitts’ (1943) proposal did not completely fit into it. Two aspects of this theory were left out.

The first was the assertion of the reticular character of the functional organization of the brain. Computationalism was univocally focusing on a linear cognitive organization – ‘input-processing-output.’ While approving of this view, McCulloch and Pitts connected it to the idea of the reticularity of the brain – a thesis inherent to their use of networks for modeling neuronal activity.

The second aspect of the 1943 McCulloch-Pitts article left out by computationalism was its thematization of mind. McCulloch and Pitts developed it along two of the main axes of traditional theory of knowledge: *rationalism*, evident in their interpretation of cognition in terms of logical computation, and *representationalism*, explicit in the idea of the intentionality of “psychic events,” due to their “inherently propositional” character. Despite this, the two cyberneticians, through their thesis of the immanence of mind in brain activity, were refusing the third pillar of classical theory of knowledge – i.e., the substance *dualism* of body and mind. Computationalism, in contrast, progressively reconstituted this dualism based on the characterization of the brain/mind relation in terms of the differentiation between computer hardware and software. In the late 1960s, this distinction was radicalized in a dichotomy through the “multiple realizability” thesis, according to which a single mental kind (a mental property, state, or event) can be realized in different physical kinds in such a way that it is essentially independent and unconstrained by the specificities of its materialization. This entailed that the computational mind could be seen as an inherently immaterial logic machine, functioning independently of the specifics of brain and body. In other words, mind could be regarded as software that runs equally well on brains and on other forms of hardware. Consequently, its exploration could be independent from the study of brain and body – a view that is incompatible with the methodological positioning of McCulloch’s experimental epistemology.

These two elements of the 1943 McCulloch-Pitts proposal – reticularity and immanence – can be recognized as two key elements of the constructivist paradigm inspired by this work. As its label suggests, this research paradigm stands as an alternative to computationalism. Constructivism rejects the traditional characterization of cognition as a representation of an independent reality, and qualifies it as an adaptive function through which living systems actively organize – construct – their world of reference (Ceruti, 1987, 2007; von Glasersfeld, 1995; Damiano, 2009).^1^

### Scaffolding for a Cybernetics of Autonomy

A few years after the publication of the 1943 article, McCulloch started to recognize the limitations of the approach. He did not simply point out that biological neurons, unlike McCulloch-Pitts neurons, can compute different Boolean functions, and work not only in series, but also in parallel. He further argued that the biological brain does not include networks like the McCulloch-Pitts ones – that is, networks structured to compute a specific Boolean function. According to him, this was because genes cannot specify the wiring of the brain, which implies that the majority of neuronal connections have to be generated by learning during interactions with the environment.

As we have 10^10^ neurons, we can inherit only the general scheme of the structure of our brains. The rest must be left to chance. Chance includes experience which engenders learning [… as …] the growing of new connections. […] Why is the mind in the head? Because there, and only there, are hosts of possible connections to be formed as time and circumstance demand. (McCulloch, 1951, pp. 85–86)

For McCulloch, this consideration expressed the need for a methodological change in his synthetic modeling approach. Instead of choosing a cognitive function and then defining the structure of a network able to realize it, the modeling had to start from networks based on random connections among artificial neurons and attempt to determine what properties and functions the networks were able to develop in interaction with an environment.

At the theoretical level, this focalization on “chance” expressed the thesis that brain connections are not exogenously determined either. Unlike a computer, the brain is not a heteronomous system built and instructed by an external constructor. The brain is an autonomous system that determines itself through a self-organization dynamic taking place within the twofold ecology of body and environment. The way to explore the brain in action experimentally, through the synthetic approach, had to entail bringing into the experimental scene the brain’s own activity of self-organization during interaction processes.

McCulloch and Pitts started to implement this change of perspective in studies exploring neurophysiological mechanisms underlying pattern recognition. The 1947 article presenting this work – *How We Know Universals* (Pitts and McCulloch, 1947) – offered new perspectives not only in histology, anatomy, and physiology (Kay, 2001), but also in synthetic modeling of brain activity. The novel approach was based on probabilistic Boolean networks, whose design took into account instability and indeterminacy of neuronal dynamics. The networks were articulated on layers of populations of neurons that computed in sets. The focus was not on the logic performances of the networks, but on the cognitive function of perception that they were modeling – the interaction between the networks and their environments.

This series of shifts in the McCulloch-Pitts cybernetics of networks – from problem solving to learning, from serial to parallel computation, from input-output to reticular organization, from digital computers to situated biological-like systems – promoted the rise of the constructivist paradigm.

### Networks as Biological Computers. Toward Constructivism

One of the earliest protagonists of this development was Heinz von Foerster, a member of McCulloch’s team at Illinois University. In the late 1950s, he founded a new laboratory there: the Biological Computer Laboratory – BCL (Müller and Müller, 2007). Its main assignment was to use artificial networks to conduct synthetic explorations of cognition able to account for the autonomy of biological systems. Von Foerster (1960, 1982) based this undertaking on a model of cognitive system that can be seen as a corrective of the computationalist one. He called it a “biological computer,” and assigned it the task of bringing into focus what the digital computer model obscured. Living systems, unlike man-made computers, are self-organizing systems, and develop cognition not as abstract problem solving, but as the inherently biological activity of surviving in an ever-changing “hostile” environment.

The theoretical core of the biological computer model can be found in a reworking of the McCulloch-Pitts thesis (cognition = computation) that amplifies the 1947 revision of cybernetics of networks. Von Foerster grounded this reformulation in a principle of continuity between life and cognition. Based on this principle, and on a literal understanding of the Latin verb “computare” as “considering things together,” he redefined computation as every operation that a living system, as a self-organizing network of processes, is able to perform to preserve itself (von Foerster, 1974). It is a reconceptualization that transfers the notion of computation from the epistemological space of abstract problem solving to that of biological adaptation. Through this notion, von Foerster designed a model that “biologizes” the McCulloch-Pitts idea of computing networks, and conveys strong versions of the immanence and reticularity theses.

Von Foerster’s interpretation of computation requires conceiving of the cognitive mind as immanent not only in brain activity, but also in the other processes instantiating biological self-organization. Along the lines sketched by McCulloch and Pitts, it portrays mind not as “a ghost in the [biological] machine,” but as the functioning biological machine itself. That is, not as a substance, but as a process: a dynamic, distributed whole, whose functions are realized through computations performed not only by neurons, but also by the other operative sub-units distinguishable in biological networks.

Correlatively, “self-referentiality” is the logical structure that von Foerster (1977) ascribed to biological computation. His idea was that, given the organizational reticularity of living systems, every computing operation that occurs in them is both the result and the starting point of another computation. In other words, all operations of a biological computer result in other operations inside its network. This insight, which von Foerster (1977) structured around Jean Piaget’s notion of “closure,” generated his main epistemological thesis. The cognitive operations of a living network do not refer to an external or transcendent reality. They are instances of “Eigen-behavior”: operations that the network, as an organized set of components, performs on itself.

This is the nucleus of von Foerster’s constructivist position, which, based on previous analyses of his work (Ceruti, 1987, 1994, 2007; Damiano, 2009), we can summarize through three theses contrasting with classical computationalism. (1) *The environment is not a reservoir of information for the biological computer.* It is an operationally independent source of energy and matter to which the network must couple to fuel its processes. (2) *The biological computer, while thermodynamically open, is informationally closed.* Its circular organization precludes the reception of exogenously defined information. It enables the system to perceive some environmental events as perturbations, and to react through self-regulation. (3) *The biological computer is a creator of information.* By associating conservative Eigen-behaviors with experienced perturbations, the systems loads perceived environmental variations with operational meanings. It treats these variations as though they were carrying information on effective self-preserving behavior for those conditions. Through its self-referential computation, it treats a perturbing environment as a meaningful world – it constructs a reality of reference for its interactions (von Foerster, 1979).

This constructivist reinterpretation of the McCulloch-Pitts cybernetics of networks positions von Foerster among the precursors of radical embodiment. His model of the biological computer gives the cognizer’s body a much stronger role than that postulated by McCulloch and Pitts. In von Foerster’s work, the body is not simply the organic support for the brain, understood as the only organ where cognitive processes take place. Identification of living systems with computing networks implies that the cognizer’s body is a “cognitive body” (Montebelli et al., 2009) – a body that, in its integrality, instantiates cognitive processes. From this perspective all mental functions – consciousness included – appear grounded in the self-referential computation of the cognizer’s body, and participating both in her self-organization and in the organization of her world.

This variant of experimental epistemology had significant impact on the rise of radical embodiment. Starting in the late 1950s, it began converging in BCL’s groundbreaking work on artificial self-organizing systems that, like Gordon Pask’s work, has been inspiring radically embodied approaches in AI since the 1990s (e.g., Cariani, 1993; Bird et al., 2003). Moreover, in the late 1960s, von Foerster’s framework began to be elaborated upon by other strands of experimental epistemology, which pioneered radical embodiment.

## The Cognitive Biology of Networks

### Autopoiesis: Metabolic Networks as Cognitive Systems

When considering the future of experimental epistemology in the 1960s, McCulloch (1961b) recognized as continuators of this research tradition the members of the team that had done, under his supervision, the neurophysiological study on frog vision resulting in the article “What the Frog’s Eye Tells the Frog’s Brain” (Lettvin et al., 1953). They included the neurobiologist Humberto Maturana, who, years later, paid homage to this legacy by using the notion of experimental epistemology to define his work in cognitive biology (Maturana, 1974). Its main result, developed by Maturana with his former student Francisco Varela, was the theory of autopoiesis, often included among the theoretical sources of radical embodiment (Clark, 1997; Di Paolo and Thompson, 2014; Ziemke, 2016).

The scientific program that Maturana and Varela (1980, 1987/1998) assigned to autopoiesis was that of addressing, in conjunction, two fundamental questions – “What is life?” and “What is cognition?” Their undertaking was based on the thesis of continuity between life and cognition, and, like von Foerster’s, it placed the emphasis on the autonomy of living systems. Their notion of autopoiesis (namely: self-production) postulated biological autonomy and defined it as the distinctive capability of biological systems to produce and maintain their material identity – themselves – through the production of their components. Based on this notion, Maturana and Varela formulated the above-mentioned principle as an equation: a (life = cognition) hypothesis that characterizes its terms as instantiations of the process of autopoiesis – metabolic self-production.

Using this framework as a reference, Maturana and Varela saw the possibility of providing a scientific definition of life and cognition. Their idea was to create a “synthetic” definition, based on a theoretical implementation of the synthetic method (Damiano, 2009; Damiano and Stano, 2018a,b). Concretely, this meant using biological insights to define an organizational mechanism able to generate the minimal form of autopoiesis (i.e., cellular autopoiesis), and then conceptually testing this mechanism’s capability to produce known living and cognitive phenomenology.

Maturana and Varela packaged this synthetic definition in the notion of “autopoietic organization,” which describes the cell’s autopoietic organization by drawing on the McCulloch-Pitts descriptive schema – the model of a network of operations realized by a population of interacting components.

[The autopoietic organization is] a network of processes of production (transformation and destruction) of components that produces the components which: (i) through their interactions and transformations continuously regenerate and realize the network of processes (relations) that produced them; and (ii) constitute it (the machine) as a concrete unity in the space in which they (the components) exist by specifying the topological domain of its realization as such a network […] (Maturana and Varela, 1980, p. 79).

This definition, meant to characterize minimal living and cognitive organization, resulted in a further “biologization” of the McCulloch-Pitts cybernetics of networks. Through the notion of autopoietic organization, Maturana and Varela elaborated on von Foerster’s identification of living networks as cognitive networks, but dropped his qualification of them as computing networks – biological computers. The description of the autopoietic processes in terms of transformation operations, with no reference to computation, was a complete rejection of the metaphor of the computer, which could still be found – although residually – in von Foerster’s work. This dismissal makes the theory of autopoiesis more than a correction of computationalism. It is a radical alternative, proposing to model basic cognitive organization not on a computer, but on a metabolic network instantiating a minimal self-individuating body.

One of the most interesting aspects of the notion of autopoietic organization is that it succeeds in capturing a characteristic trait of cellular dynamics: the fact that the system’s components continually change, while the system itself, as a relational unity of components, remains. This notion describes cellular metabolism as a dynamic, reticular chain of operations of elementary transformations that, by activating the synthesis or destruction of its material components at every step, generates a twofold effect. On one side, it produces changes in its own materialization – i.e., in the autopoietic system’s material structure. On the other side, it triggers the re-instantiation of the functional relations that constitute it as a network of operations and regenerate it. This is a thematization that Maturana and Varela implemented through the complementarity of “organization/structure,” grounding a new approach to characterizing the mind/body relation.

The notion of autopoietic organization can be recognized as sketching the preliminary lines of a theory of mind that diverges radically from the classical view of an entity essentially independent from its materialization. What Maturana and Varela described through this notion is a cognitive organization that constantly generates the process of its material realization, and is constantly regenerated by it. From this perspective, mind, as a cognitive organization, and body, as its physical realization, cannot be considered as two different substances, independent of each other. They appear as two inseparable aspects of the same process – biological self-production.

This is a reworking of the McCulloch-Pitts point of view. Mind is a process, not a substance. It is a dynamic, collective whole distributed, first and foremost, across the metabolic operations producing the biological body. Mind is not the “ghost in the [biological] machine,” but the dynamic biological machine itself – which, for Maturana and Varela, could not be described as a computer.

Autopoietic machines do not have inputs or outputs. They can be perturbed by independent events and undergo internal structural changes which compensate these perturbations. (Ibid.:81)

Through their expansion of the McCulloch-Pitts theses of reticularity and immanence, Maturana and Varela developed a constructivist view convergent with von Foerster’s in recognizing the self-referentiality of biological cognition and the related inaccessibility of an “absolute reality.” They structured this view around the notion of “structural coupling,” which inter-relates the autopoietic system and its environment in a dynamics of reciprocal perturbations and self-determined regulations. This notion defines the continuance of the system-environment coupling as the condition for maintaining autopoiesis, and assigns biological cognition the task of meeting this condition. This relies on self-regulatory activity through which the autopoietic system, as it produces itself interacting with its environment, “brings forth a world.” That is, it loads perturbing events with operational meanings supporting its effective action in its domain of existence (Maturana and Varela, 1987/1998, p. 26).

In their work together, Maturana and Varela argued strongly for the capability of their theory to synthetically explain human cognitive phenomenology, but they did not model human cognition. A synthetic approach to modeling it was developed by Varela independently.

### Autonomy: Networks as Emergent Selves

In the late 1970s, Varela started an individual research path, parallel to the one with Maturana and explicitly developed as a strand of experimental epistemology (Varela, 1986).

One of Varela’s priorities was to extend the organizational modeling of cognitive systems that autopoiesis began at the cellular level. His aim was to create a model able to bring into research focus, at all relevant levels of description, the relationship between autonomy and cognition – between self-constitution of a cognitive system and constitution of its world of reference. To this end, Varela engaged in synthetic modeling of the organization shared by all autonomous systems. The resulting notion, introduced in *Principles of Biological Autonomy* (1979) as “organizational closure,” relaunched the cognitive network schema in a variant that hybridized its previous versions – McCulloch and Pitts’, von Foerster’s, and the autopoetic one. Varela combined them with the goal of dismissing both the cybernetic concept of computation and the autopoietic reference to the (minimal) biological level. What he articulated is a general formulation of the Piagetian concept of closure, through which he intended to capture the general form of the circularity that experimental epistemology had progressively recognized as the basic organizational trait of autonomous systems.

[…] Autonomous systems are organizationally closed, that is, their organization is characterized by processes such that (1) the processes are related into a network, so that they depend recursively on each other in the generation and implementation of the processes themselves; and (2) constitute the system as a recognizable unit in the space (domain) in which the process exists. [...] The processes that specify a closed organization can be of any type and take place in any space defined by the properties of the components that constitute the process. (Varela, 1979, p. 55)

Based on this new notion of network, Varela’s model could be used to characterize as autonomous systems all structures generated by interdependence between processes. It could synthetically define as systems endowed with a certain degree of autonomy all units that, deriving from reticular co-dependence, do not work as computing input-output devices, but generate operational meanings for perturbations.

[...] For such systems all the apparent informational exchanges with the environment will, and can only be, treated as perturbations within the process that defines their closure [...]. (Ibid.:59)

In his writings, Varela (1991, 1995b) gave this model two working definitions. The first, “selfless self,” emphasizes the divergence of Varela’s “cognitive self” from the classical notion of subject. This definition reframes the autopoietic thesis according to which the subject of cognitive processes goes beyond the Cartesian alternative between two substances – an immaterial cognitive organization and a material aggregate. The “selfless self” is not a substance. It emerges from the dynamic entanglement between (immaterial) organization and the (material) structure of autonomous systems. It is distributed across the continuous process through which components are dynamically organized in a whole, and this whole conservatively interacts, as a coherent unit, with its environment by regulating the behavior of its components. This view is stressed in the 1995 definition of “emergent self,” which refers to the capability of elementary co-dependence to produce a level of organization qualitatively different from that of its constituents. It outlines a cognitive system that, as an integrative unit formed by reticular connections among elementary operations, is inherently open to developing higher-level reticular connections, allowing it to participate in higher-level cognitive units.

This theoretical construction further extends the McCulloch-Pitts view of mind. According to Varela’s model, mind is a plural process. It is a collective entity that is distributed among the interactions between different organizational levels of autonomous systems, and can participate in creating new levels. It is an intrinsically polycentric, metamorphic entity, which, if we interpret Varela’s theory of autonomy literally, is immanent not only in biological self-production, but also in other forms of autonomous systems’ self-constitution – that is, in the plurality of organizational closures and related forms of cohesion that science can individuate at its different levels of inquiry. In fact, from 1979 on, Varela’s definitions of the cognitive self did not include inherent references to the biological domain.

An underlying circular process elicits an emerging coherence and this is the cognitive self at that level. (Varela, 1995b, p. 193)

This variant of the idea of cognitive network can be recognized as the basic descriptive tool through which Varela developed his radical embodiment theory – enaction.^2^

## The Cognitive Science of Networks

### Radicalizing McCulloch’s “Embodiments of Mind”

One of the most advanced versions of Varela’s enactive theory of mind can be found in his 2001 article with Evan Thompson (Thompson and Varela, 2001). This work, entitled *Radical Embodiment*, makes it evident that the Thompson-Varela attempt to overcome dualism does not rely on the “neuro-anatomical solution” typical of mainstream embodied cognitive science. Thompson and Varela developed a “neuro-dynamic solution,” which locates the mind not in the cerebral platform, but in the processes defining the nervous system’s participation in human cognition. In their article, they described these dynamics as “cycles of operations” generating the three forms of co-evolution supporting human cognition: the couplings between, respectively, nervous system and body, organism and environment, and agent and other agents.

Through the first of these processes, Thompson and Varela grounded the embodied mind in the cyclical operations through which the nervous system regulates the complex dynamics of the human organism’s self-production. Building on Varela’s previous works, *Radical Embodiment* characterizes the human body as a set of closed networks interconnected with each other and with the nervous system. The Varelian model of the emergent self can be recognized in action. It can be seen as the key to interpreting all the networks constituting the human body as enmeshed autonomous systems. From the 1990s on, Varela conceptualized them as co-evolving “cognitive selves” recognizable in “cells, tissues, organs [...]”, and in the “bio-mechanical networks, bio-chemical networks, physiological networks [...]” of the human body. Drawing on Paul Weiss and Gregory Bateson’s idea of ecologies of mind, Varela (1991) thought of these somatic networks as “a meshwork of selfless selves” coupled with the nervous system.

This is a reworking of the autopoietic characterization of the body as a cognitive network (of networks), on the basis of which Varelian enaction deepens McCulloch’s idea of “embodiments of mind.” It pushes experimental epistemology to move from McCulloch’s “mind is in the head” thesis to a view that, in line with autopoiesis, locates mind “not in the head” (Varela, 1999), but, first and foremost, among the rhythms and patterns of human body self-production.

Thompson and Varela (2001) further redefined McCulloch’s location of mind based on the description of the dynamics through which the nervous system couples the human organism and the environment. They characterized it as “sensorimotor coupling,” reframing the constructivist modeling of the nervous system in terms of a closed network that interconnects *sensorium* and *motorium*, and maintains their coordination via self-regulation. This is the framework, introduced by pioneers of constructivism such as Piaget and von Foerster, which rejects the traditional hypothesis of a representational phase between perception and action. The description of the nervous system in terms of the sensorimotor closure of the human organism replaces traditional representations by neuronal patterns of self-regulation, that is, patterns of neuronal activity that associate sensorial perturbations with actions favoring the organism’s stability. It is an option that supports the constructivist view of human cognition according to which the objects we deal with cognitively, far from being predefined external entities that we represent internally, are “tokens of Eigen-behaviors” (von Foerster, 1977). In the 1990s, Varela and Thompson, with Eleanor Rosch, conveyed this perspective in the notion of enaction as “embodied action” that “brings forth a world.” In short, the neuronal network associates sensorial perturbations with patterns of self-regulation that project, on the perceived aspects of the environment, objects expressing the cognizer’s “readiness for action” – her contextual possibilities of action, defined by her body structure (Varela et al., 1991).

This perspective radicalizes Varela’s (1999) “mind is not in the head” thesis. According to this view, mind, far from being located in the *intra-*individual space, is immanent in the co-specification dynamics that correlates somatic networks, the neuronal network and the environment. Its place is the coupling that links not only brain and body, but also organism and environment, and, thus, the cognizer and other agents. As the proponents of experimental epistemology saw, mind is a process. Yet, it is not a process confined to the brain or the body. Being immanent in neuronal activity, mind is distributed in the interaction through which brain, body and environment interdependently define their patterns of activity. It is by interconnecting brain, body and environment – other agents included – in a continual process of co-transformation that mind instantiates human cognitive processes.

### Neuronal Networks and Self

This view of mind as dynamic interconnection of brain, body and environment is reflected in Varela’s studies on neural correlates of consciousness. In the 1970s, Varela began working on characterizing the neuronal ensembles supporting the agent’s sensorimotor coupling in terms of self-organizing behaviors. The key idea was that of a quick, flexible, functional coordination between distant areas of the brain (i.e., large-scale “neuronal integration”) mediated by resonances among neuronal oscillations – a “synchronization” of their phase of oscillation connecting neurons in temporary coherent collective units (Varela et al., 2001). Varela described these functional units as neuronal assemblies that “interpret” the organism-environment coupling, and, on this basis, orient its future (Varela, 1995a). This is another application of his model of the network, which characterizes the processes of neuronal integration as dynamics generating transitory autonomous systems – organizational closures that instantiate emergent selves on the micro-temporal scale (Damiano, 2009).

Varela developed this view explicitly in the 1990s, defining neuronal ensembles as “micro-identities”: transient selves expressing *readiness for action* – contextual possibilities of action grounded in the agent’s body. Their instantiation, through neuronal integration, determines the specific way in which, at a certain moment, the cognizer brings forth her world. When a micro-identity is self-constituted, via a process of self-organization triggered by exogenous or endogenous perturbations, a related “micro-world” arises at the level of subjective experience. The agent is immersed in a new situation, defined by the *readiness for action* that her temporary micro-identity supports.

You put your hand into your pocket [...]. Breakdown: you stop, your thoughts are muddled, your emotional tonality shifts. [...] A new world emerges: you see clearly that you left your wallet in the store where you just bought cigarettes. Your mood shifts now to one of concern [...], your readiness for action is now to quickly go back to the store [...].^3^ (Varela, 1992, p. 11)

This can be seen as an innovative variant of McCulloch and Pitts’ modeling of neuronal activity, used by Varela to propose a radical alternative to their original idea of consciousness as an objective, “from nowhere” representation of the system-environment relation. Varela’s characterization of neuronal activity reframes the McCulloch-Pitts descriptive pattern of the network, and thereby grounds, in neuronal dynamics, a highly situated, perspective-based form of conscious experience. Indeed, what Varela defined, through the hypothesis of a punctuated succession of micro-identities and micro-worlds, was an intrinsically situated conscious agent whose point of view is continually reconfigured by variations in situational context and attentional focus.

Based on experimental insights, this radically embodied approach to conscious experience was reproposed in the 2001 Thompson-Varela article. It was a rigorous reframing, with the ambition of giving the self a new status, beyond the alternative between by-product of brain activity and substantial subject of experience. In line with the general methodological positioning of experimental epistemology, Thompson and Varela portrayed the self as a legitimate, non-epiphenomenal object of empirical and quantitative inquiry, synthetically explainable in terms of mechanisms – the mechanisms of neuronal integration. It is on this basis that, converging with the non-substantialist approach inherited from its tradition, Varelian enaction challenges the Cartesian image of the self. It proposes to conceptualize the self no longer as a permanent, consistent center of conscious experience, but as a process – notably, a contingent, discontinuous, highly distributed process.

The contingency of the enactive self is upheld in its theoretical characterization, which correlates it with non-linear self-organization dynamics that reflect structural and historical specificities of the plural coupling of the nervous system. Discontinuity is inherent to this process, which structures and de-structures neuronal ensembles – that is, transitory *readiness for action* defining the (micro-)identities of the self – in reaction to breakdowns that punctuate the interactions of the nervous system within its twofold ecology – organism and environment. The distributed character of the self stems from this co-evolution, which establishes a radical interdependence between the changes in the self’s identity and the modifications intervening in the patterns of activity of the somatic networks, environment and agents with which the cognizer interacts.

From this perspective, the self of conscious experience appears as an event – a brain-body-environment-others event. Correlatively, consciousness, as a process arising in the complex of the radically embodied mind, appears to cut across the traditional partitions of cognitive science, thereby bringing into question not only its classical notions and theses, but also the standard individuation of its relevant research objects.

This view influenced recent frontier research on mind and consciousness, such as the pattern theory of self (Gallagher, 2013), neuro-scientific characterizations of self-related processes (Antonova and Nehaniv, 2018), and the self-organizational theory of machine consciousness (Tani, 2017). By developing the framework of Varela’s enaction, these works still draw on the lines of McCullochian experimental epistemology, and implicitly grant to this research tradition further impact on the advancement of scientific exploration of mind and consciousness.

## The Heritage of Experimental Epistemology

**FIGURE 1:**
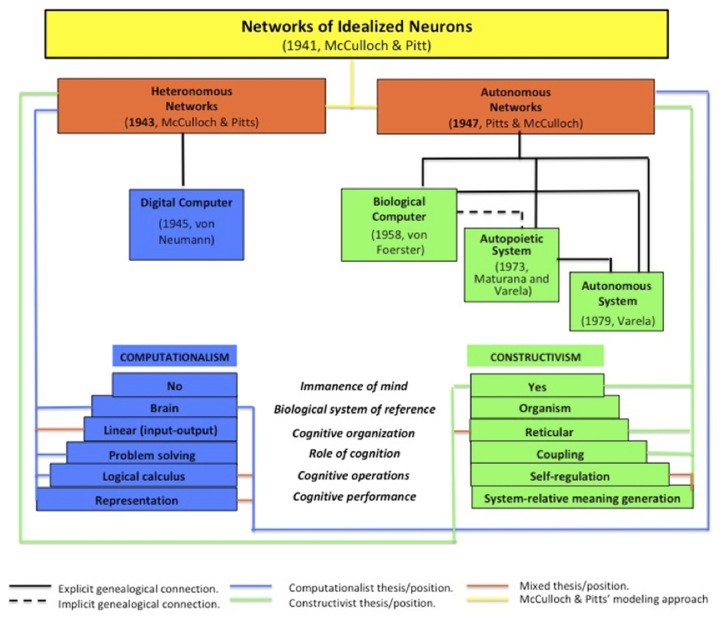
The genealogy of McCulloch and Pitts’ cybernetics of networks.

One of the most interesting aspects of experimental epistemology is the variety of its scientific production, spanning from classic computationalist to radical embodiment stances (Figure 1). This diversity, as we have shown, is crosscut by significant convergences in research approaches. They rely on common theoretical and methodological axes of development, along which the strands of this tradition have articulated their respective contributions to the shared objective of deepening the scientific understanding of “embodiments of mind.” Indeed, the evolution of experimental epistemology, from McCulloch-Pitts computational cybernetics to Varelian enaction, can be read as the ongoing elaboration of a research strategy able to tackle, in a scientific manner, a classical epistemological issue – namely, how the body is involved in mental processes.

Today this issue is still open, and appears far from being solved. As is clear from the literature, three decades after the establishment of the new “body-centered” cognitive science and AI, there is still no consensus on how to interpret its main thesis, according to which the body is crucial to cognition (Di Paolo and Thompson, 2014). Moreover, many of the dissonant formulations of this thesis are currently recognized as local, *ad hoc* redefinitions of computationalist dualism, which portray the biological body as a “a physical container” whose role is to allow the classical “computational mind to interact with its environment through sensors and actuators” (Ziemke, 2016). Therefore a growing number of contemporary specialists question the innovativeness of the “embodiment turn” in cognitive science and AI, and consider it more as an adjustment of computationalism than the rise of a novel paradigm (e.g., Shapiro, 2007; Wheeler, 2014; Ziemke, 2016). In this sense, it appears that one of the main requirements that the embodied approach to cognitive science and AI has to meet, to fully establish itself as an alternative, is to develop non-computationalist (non-dualist) accounts for the role(s) of the body for cognition.

Agreeing with these analyses, we think that experimental epistemology can further contribute to the advancement of cognitive science and AI specifically with regard to this issue. We convey this tradition’s heritage for future research on the embodied mind in three guidelines proposing an alternative to the computationalist approach, and its contemporary variants, to investigating and characterizing the role(s) played by the body in cognitive processes.

### (1) Recognizing the Whole Body as a Cognitive System

The body’s organization, describable as a network (of networks) of processes of transformation of components, instantiates the organism as an inherently cognitive system, which, while producing itself, conservatively modulates its coupling with the environment, and thereby expresses cognition as effective operativeness in its domain of existence. This perspective dismisses computationalist dualism by fully identifying the biological body’s organization as a cognitive organization, and characterizing it as a network of operations entangled with, and thus inseparable from, its material realization.

### (2) Exploring All Cognitive Functions – Consciousness Included – As Inherently Dependent on the Body, and Reflecting Its Organizational Circularity

There are two main implications flowing from characterizing the body’s organization as a cognitive organization grounding all cognitive functions. The first is the indispensability of the body, flesh and blood, for the expression of cognitive functions, included high-level ones. The second is the self-referentiality of biological cognition, which, due to the reticular organization of the body and its constitutive networks, is made of operations that refer not to an external or transcendent reality, but to other cognitive operations. What they instantiate is self-regulation “Eigen-behaviors,” which generate operational meanings for perturbing events – “bring forth” a meaningful cognizer-relative world.

### (3) Synthetically Investigating How a Network of Processes of Transformation of Components Can Instantiate Continuity Between Matter, Life and Cognition

Today embodied AI concentrates mainly on the biological body’s structure, and explores how certain cognitive tasks can be accomplished through interactions between body and environment without requiring (or reducing the involvement of) high-level cognitive processes. This work, as it produces hardware that performs cognitive tasks, can be seen as a first, significant step toward overcoming the computationalist hardware/software dichotomy (Pfeifer and Scheier, 1999). Yet, to fully model the role(s) of the biological body in cognition, and overcome the classical body/mind dichotomy, embodied AI has to take a further step. It has to focus on the biological body’s organization through synthetic modeling of how its reticular dynamics, while generating a self-producing unit of components, instantiates its cognitive coupling with the environment. As argued in (Damiano and Stano, 2018b), this research topic, which AI specialists approach rarely and mainly theoretically, could be developed experimentally, in particular through cross-fertilization between embodied AI and synthetic biology based on chemical Boolean networks. Although a full “wetware modeling” of the biological body’s organization, through this kind of network, currently appears out of reach, any intermediate model would deepen the synthetic understanding of embodied cognition, and could lead to the creation of new forms of (minimal) cognitive bodies, different from the biological archetypes.

The development of the tradition of experimental epistemology suggests these guidelines should be implemented through an inherently plural approach, based not on the mutual exclusion, but on the coordination of different disciplines and methods, and even the co-evolution of alternative (reciprocally perturbing) research paradigms, as a strategy to multiply access to the embodied mind. This might be one of the messages conveyed in the plural form of the McCullochian label “embodiments of mind.”

## Author Contributions

LD and MC equally contributed to this work in a substantial and direct way, and approved it for publication.

## Conflict of Interest Statement

The authors declare that the research was conducted in the absence of any commercial or financial relationships that could be construed as a potential conflict of interest. The handling Editor declared a shared affiliation, though no other collaboration, with one of the authors MC.

## Footnotes

1 With our definition, and the following analysis of experimental epistemology’s constructivist developments, we refer in particular to the strand of constructivism that Alexander Riegler calls “biological constructivism” (Riegler, 2012).

2 According to current mappings (e.g., Di Paolo and Thompson, 2014), radical embodiment approaches include Francisco Varela’s enaction and its developments based on Varela’s collaboration with Evan Thompson (Varela et al., 1991; Thompson and Varela, 2001).

3 The translation is ours.
